# Calcium Sulphate/Hydroxyapatite Carrier for Bone Formation in the Femoral Neck of Osteoporotic Rats

**DOI:** 10.1089/ten.tea.2018.0075

**Published:** 2018-12-03

**Authors:** Aurimas Širka, Deepak Bushan Raina, Hanna Isaksson, K. Elizabeth Tanner, Alfredas Smailys, Ashok Kumar, Šarūnas Tarasevičius, Magnus Tägil, Lars Lidgren

**Affiliations:** ^1^ Department of Orthopedics and Traumatology, Lithuanian University of Health Sciences, Kaunas, Lithuania.; ^2^ Department of Clinical Sciences Lund, Orthopedics, Faculty of Medicine, Lund University, Lund, Sweden.; ^3^ Department of Biomedical Engineering, Lund University, Lund, Sweden.; ^4^ School of Engineering, University of Glasgow, Glasgow, United Kingdom.; ^5^ Department of Biological Sciences and Bioengineering, Indian Institute of Technology Kanpur, Uttar Pradesh, India.

**Keywords:** osteoporosis, bisphosphonates, bone morphogenic protein-2, bone regeneration, femoral neck, ceramics

## Abstract

This study investigated bone regeneration in the femoral neck canal of osteoporotic rats using a novel animal model. A calcium sulphate (CS)/hydroxyapatite (HA) carrier was used to deliver a bisphosphonate, zoledronic acid (ZA), locally, with or without added recombinant human bone morphogenic protein-2 (rhBMP-2). Twenty-eight-week-old ovariectomized Sprague–Dawley rats were used. A 1 mm diameter and 8 mm long defect was created in the femoral neck by drilling from the lateral cortex in the axis of the femoral neck, leaving the surrounding cortex intact. Three treatment groups and one control group were used: (1) CS/HA alone, (2) CS/HA + ZA (10 μg) (3) CS/HA + ZA (10 μg) + rhBMP-2 (4 μg), and (4) empty defect (control). The bone formation was assessed at 4 weeks post surgery using *in vivo* micro computed tomography (micro-CT). At 8 weeks post surgery, the animals were sacrificed, and both defect and contralateral femurs were subjected to micro-CT, mechanical testing, and histology. Micro-CT results showed that the combination of CS/HA with ZA or ZA + rhBMP-2 increased the bone formation in the defect when compared to the other groups and to the contralateral hips. Evidence of new dense bone formation in CS/HA + ZA and CS/HA + ZA + rhBMP-2 groups was seen histologically. Mechanical testing results showed no differences in the load to fracture between the treatments in either of the treated or contralateral legs. The CS/HA biomaterial can be used as a carrier for ZA and rhBMP-2 to regenerate bone in the femoral neck canal of osteoporotic rats.

## Introduction

Osteoporosis and fragility fractures are increasing with the increase in age and are only partly halted by primary and secondary national prevention programs.^1^ More than 8.9 million osteoporotic fractures occur annually worldwide, with approximately one-third in Europe.^2^ The direct cost of managing osteoporosis was estimated at €37 billion, but if quality-adjusted life-years lost were considered, the overall cost amounted to €98 billion in Europe in 2010.^2^ Due to the aging population, fragility fractures are predicted to increase further in the next decade.^2^ The life expectancy in hip fracture patients decreases by nearly 25% when compared to age- and sex-matched populations.^3^ One fifth of all fragility fractures occur in the hip, with an almost equal ratio in the cervical and trochanteric region.^2^

Primary and secondary prevention methods evaluated for osteoporosis include calcium and vitamin D supplements, bisphosphonates, parathyroid hormone and its analogues, and biological drugs targeting resorption (Denosumab, anti-RANKL).^4^ Zoledronic acid (ZA) is a bisphosphonate and has been shown to reduce the risk of hip fracture by 41% in postmenopausal women.^5^ However, systemic usage of ZA is reported to have side effects, including myalgia^6^ and osteonecrosis of the jaw, with prolonged use.^7^ The interest in osteoporosis therapy is declining, in particular for secondary fracture prevention where the risk of subsequent hip fractures following a first fracture is high.^8^ This is to some extent based on reports of long-term adverse effects with systemic bisphosphonates causing atypical fractures and lack of patient adherence to taking bisphosphonates, with half of patients stopping treatment during the first year.^9^ Thus, there is a need for novel preventive treatment modalities, especially as major osteoporotic fractures *per se* are linked to increased mortality.^3^

Bone morphogenic protein-2 (BMP-2) induces osteogenic differentiation of mesenchymal cells and preosteoblasts into mature osteoblasts.^10,11^ Several studies have indicated that sustained and controlled release of BMP-2 over a longer period of time is required for optimal bone regeneration.^12^ The currently approved carriers for local delivery of recombinant human BMP-2 (rhBMP-2) provide only a burst release.^13^ Moreover, rhBMP-2 induces premature bone resorption by also stimulating osteoclasts,^14,15^ which in turn can be controlled by co-delivery of ZA.^11,16,17^ To investigate this approach, the authors developed an injectable calcium sulphate/hydroxyapatite (CS/HA) based biomaterial, which has been approved for clinical use in Europe.^18,19^ The powder phase of the CS/HA biomaterial is premixed at a ratio of 60:40 (CS:HA) by weight. The powder is mixed with a liquid phase consisting of an iodine-based contrast agent to allow radiographic visualization. After mixing with an aqueous solution, the CS hemi-hydrate particles change state to the dihydrate, causing the biomaterial to set into a hard mass, wherein HA particles are embedded in the composite material. The CS phase dissolves in less than 2 months,^20^ leaving behind a porous osteoconductive matrix of HA as a scaffold for bone cells to grow on. In a previous study, local co-delivery of rhBMP-2 and ZA using the CS/HA biomaterial as carrier led to a significant increase in bone formation in an abdominal muscle pouch model when compared to BMP-2-only group.^16^ Other studies have shown that when ZA was added to the carrier in a tibia defect model in rats, more newly formed bone remained within the defect compared to when the material was used alone.^21,22^

This study created a novel animal model to investigate the process of bone regeneration in the femoral neck canal in osteoporotic rats. Second, the study investigated whether a CS/HA carrier delivering ZA or a combination of ZA and rhBMP-2 can improve bone regeneration in the femoral neck of osteoporotic rats with the long-term goal of preventing future osteoporotic fractures. It was hypothesized that (1) HA would provide an osteoconductive scaffold for bone formation and controlled delivery of both rhBMP-2 and ZA by interacting with them; (2) the fast resorbing CS would increase scaffold osteoconductivity and provide release of unbound rhBMP-2 and ZA; (3) rhBMP-2 would lead to differentiation and proliferation of progenitor cells; and (4) ZA could counteract premature bone resorption, leading to increased net bone formation.

## Methods

### Sample preparation and experimental groups

Animals were randomly allocated to the following groups (*n* = 12/group): (1) CS/HA (CS/HA in the ratio of 60:40 w/w), (2) CS/HA + ZA (10 μg), (3) CS/HA + ZA (10 μg) + rhBMP-2 (4 μg), and (4) empty defect (drilled but no filling). For the CS/HA group, the ceramic components were mixed without the addition of any bone active molecules. All animals with CS/HA treatment received 116 mg of the CS/HA powder, and the liquid-to-powder ratio remained unchanged (0.43 mL–1 g powder). The addition of ZA and rhBMP-2 was performed by adding a known amount of ZA solution or the combination of ZA and rhBMP-2 solution to the ceramic powder, followed by rigorous mixing. The doses of rhBMP-2 and ZA were chosen based on a previous study using the CS/HA biomaterial.^22^ After mixing, the material was transferred into a 1 mL graded syringe. Equal portions of the sterile paste were delivered onto a sterile dish, and each portion was impacted into one animal, thereby controlling the amount of the CS/HA biomaterial and respective drugs. The biomaterial was allowed to reach a suitable viscosity for impaction before implantation to allow for complete filling of the defect without any backflow of the material.

### Animal model and surgical procedure

**FIG. 1.**
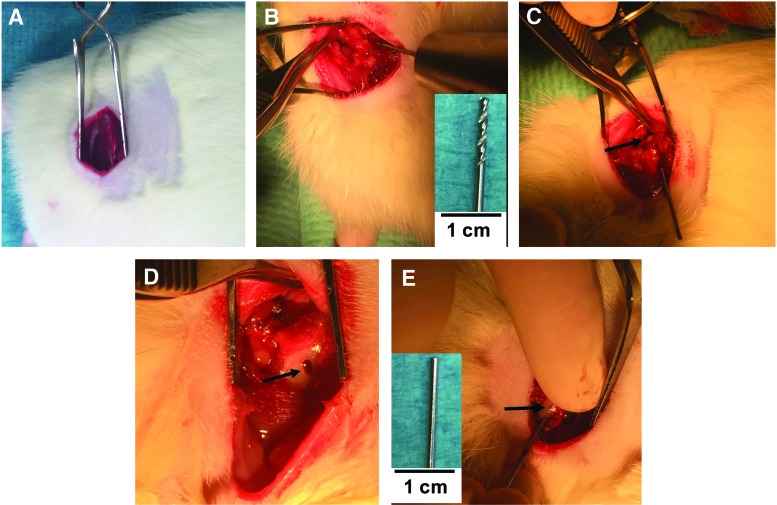
Surgical procedure. **(A)** The exposed site of incision. **(B)** The bone being drilled at the crest of the femur using a posterior approach with the drill bit used to create the defect (*inset*). **(C)** The position of the drill bit after completion of the drilling, and the *black arrow* points at the femoral head. **(D)** The point of entry in the cortical bone. **(E)** A piece of CS/HA biomaterial (*black arrow*) being impacted into the defect using a mandrel. *Inset* in **(E)** shows the mandrel used for impaction. CS, calcium sulphate; HA, hydroxyapatite. Color images available online at www.liebertpub.com/tea

Ovariectomized (OVX) female Sprague–Dawley rats of 28 weeks age at surgery with an average weight of 442 ± 29 g were used. OVX was performed at 12 weeks of age (Janvier Labs, Le Genest-Saint-Isle, France). The animals were left for 16 weeks to develop osteoporosis based on a previous study.^23^ Animals were anesthetized using an intraperitoneal injection of ketamine (90 mg/kg; Apoteket AB, Karlstad, Sweden) and xylazine (9 mg/kg; Apoteket AB). An intramuscular injection of antibiotic solution (Streptocillin Vet; Apoteket AB) was also given before surgery. The right leg was shaved and disinfected, and a 1.5 cm straight posterolateral incision was made along the greater trochanter. The muscles were bluntly dissected and the leg internally rotated until the posterior short rotators were exposed. Tenotomy of the short rotators was performed, and the posterior capsule was reached for visualization but left intact. A 1 mm drill was used to create a critical defect in the femoral neck canal without fluoroscopic guidance, with the entry point behind the crest of the greater trochanter toward the superior-lateral part of the femoral head (Fig. 1). Drilling was stopped at the base of the sub-capital zone, producing a defect depth of approximately 8 mm (Fig. 1). At this point, the material based on each treatment group mentioned above was impacted into the femoral canal using a 1 mm mandrel until the whole canal was filled with the material. Filling was made in a similar fashion in all animals. The left leg was left intact and used as a paired control. After wound closure, a single subcutaneous injection of buprenorphine (0.04 mg/kg; Temegesic^®^; Apoteket AB) was given post surgery for pain control.

To establish the degree of osteoporosis, another three SHAM-operated and four OVX animals were used as controls, with no defect created in the femoral neck canal. SHAM animals underwent a bilateral skin incision in the lower abdomen at the age of 12 weeks, but no OVX were made. The OVX animals had their ovaries removed at 12 weeks. The animals (three SHAM and four OVX) were sacrificed at the same time as the experimental animals. The proximal tibiae were subjected to micro computed tomography (micro-CT), and trabecular bone analysis was performed ∼1.5 mm distal to the tibial growth plate, with a region of interest (ROI) extending 150 slices distally (2.25 mm in total).

### *In vivo* micro-CT at 4 weeks

The animals were anesthetized using a mixture of isoflurane (4%), oxygen, and nitrous oxide at a flow rate of 0.4 L/min. Once asleep, the animals were moved to an animal holder of the micro-CT scanner (NanoScan; Mediso Medical Imaging Systems, Budapest, Hungary), and the isoflurane was reduced to 2%. Circular micro-CT scans were taken with 480 projections and medium zoom at 65 kV voltage and 1300 ms exposure time, and the images were post-reconstructed to a voxel size of 20 μm. Micro-CT imaging at the 4-week time point was performed only on the defect side.

### Sacrifice and femur harvesting at 8 weeks

The animals were sacrificed using carbon dioxide asphyxiation 8 weeks post surgery. Both femurs (defect side and contralateral) were harvested, wrapped in saline soaked gauze, and placed in 5 mL Eppendorf tubes. All analyses were performed on both the defect and contralateral legs. Freshly harvested specimens were subjected to micro-CT and thereafter frozen at −20°C until biomechanical testing.

### *Ex vivo* micro-CT at 8 weeks

The samples in the CS/HA group (*n* = 11) and the other groups (*n* = 12) were scanned at an operating voltage of 65 kV, exposure time of 1300 ms, maximum zoom, with 480 projections in circular scan mode using a NanoScan micro-CT scanner (Mediso Medical Imaging Systems). The images were post-reconstructed to a voxel size of 10 μm.

### Micro-CT image processing and analysis

**FIG. 2.**
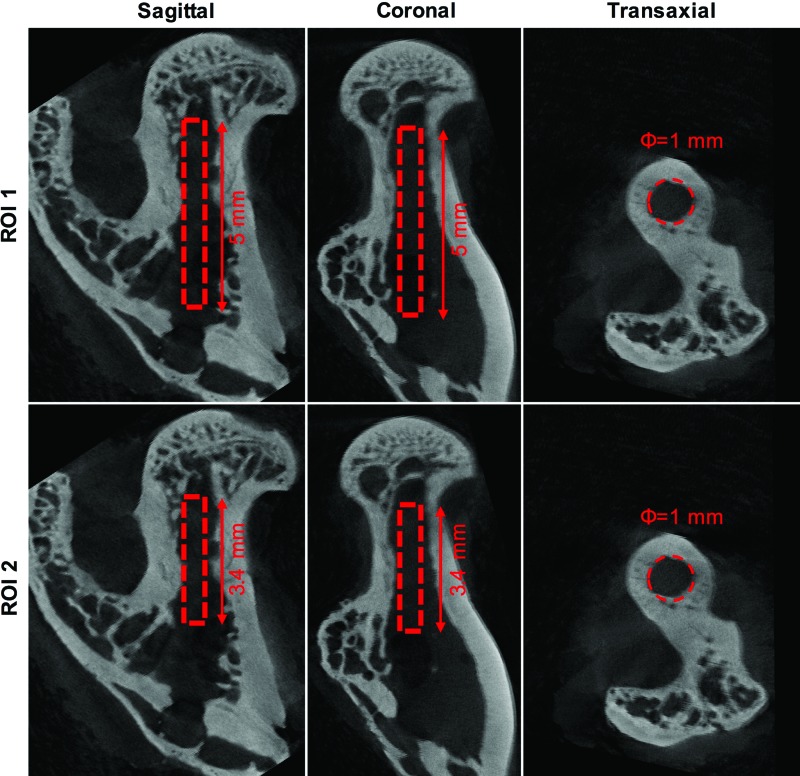
Micro-CT ROI selection for quantification. *Top panel* shows the dimensions used for micro-CT analysis in ROI 1, while the *bottom panel* shows the dimensions used for ROI 2. The *above* image is only for representation and belongs to the empty group. Micro-CT, micro computed tomography; ROI, region of interest. Color images available online at www.liebertpub.com/tea

All images from the 4- and 8-week time points were analyzed according to the same protocol. Images were post-reconstructed using Nucline software (inviCRO, Boston, MA) using a RAMLAK filter with a 100% cut-off. Images were realigned using Data Viewer (v.4; Skyscan, Kontich, Belgium) to obtain a straight rectangular defect in the sagittal and coronal views. Trans-axial slices revealed a circular defect starting from the base of the sub-capital zone down to the point of entry in the femur. The analysis was performed on the trans-axial slices using CTAn v1.9.1.0 (Skyscan) and two different ROIs were used (Fig. 2). In both ROI 1 and ROI 2, the diameter of the ROI was 1 mm, corresponding to the diameter of the drill bit. ROI 1 contained the entire defect, starting at the base of the sub-capital zone to the point of entry in the posterior cortex. The defect depth was 5 mm, which is less than the original defect, to exclude the cortical bone at the point of entry and any remaining old trabecular bone near the sub-capital zone. ROI 2 contained only the femoral neck canal region, which started at the base of the sub-capital zone to the end of the femoral neck canal (depth 3.4 mm). Images were thresholded using a lower gray scale value of 90 and an upper gray scale value of 255. This means that both bone and some remnants of the CS/HA material were identified as mineralized tissue. Mineralized volume/tissue volume (MV/TV%) was used as the primary outcome variable of the micro-CT analysis. The *in vivo* and *ex vivo* scans were performed using different imaging protocols, which were optimized for each condition. Thus, a direct comparison of MV/TV% at 4 and 8 weeks was not possible.

### *Ex vivo* biomechanical testing of the femoral neck

The bones were thawed at room temperature before performing the biomechanical testing, and care was taken to keep the samples moist until testing. All bone samples for mechanical testing (*n* = 9/group) were cut at the mid-diaphysis to ensure a stable grip during the testing. The bone specimen was kept in position using a cylindrical holding device mounted on an adjustable stage to achieve a femoral shaft angle of 9°, with the vertical plane as described by Hessle *et al.*^24^ Using a mechanical testing machine (Instron^®^ 8511.20 biaxial load frame connected to a MTS FlexTest 40 Controller, MTS TestSuite Multipurpose Elite Software) and a 250 N load cell, the femoral head was loaded using a flat-end indentation device at a displacement rate of 1 mm/s until the fracture occurred. The experimental setup is shown in Supplementary Figure S1 (Supplementary Data are available online at www.liebertpub.com/tea). The peak load to fracture was recorded for each specimen and compared between the groups as well as to the contralateral side. Moreover, the ratio of the peak force between the defect leg and contralateral leg was calculated. The location of the fracture site (lateral vs. sub-capital) was also noted.

### Qualitative histology

Bone specimens (*n* = 2/group) that were not mechanically tested were thawed to room temperature and fixed in neutral-buffered formalin overnight followed by decalcification using ethylenediaminetetraacetic acid (10% w/v, pH 7.2) for 4 weeks. Post decalcification, specimens were dehydrated and infiltrated with paraffin overnight. Finally, the specimens were embedded in paraffin and cooled at 4°C overnight before sectioning. All specimens were cut using a microtome (Thermo Fisher Scientific, Waltham, MA) to a thickness of 5 μm. Slides were deparaffinized, rehydrated, and stained with hematoxylin and eosin using standard staining techniques. The stained slides were scanned on a high-resolution slide scanner (Hamamatsu Photonics, Hamamatsu, Japan) before analysis.

### Animal ethics statement

All animal experimentation was approved by the Swedish Board of Agriculture (animal ethics permit no. M 128-16). Animals had free access to food pellets and drinking water.

### Statistics

IBM SPSS Statistics for MacOS v24 (IBM Corp., Armonk, NY) was used for statistical analysis. Distribution of data was tested using the Shapiro–Wilk test (on residues) and by looking at the spread of the data. Normally distributed data were tested using analysis of variance (ANOVA). Homogeneity of variances were tested using Levene's test, and in the case of nonhomogeneous variances, ANOVA with Games Howell *post hoc* test was used, while homogenous variance data sets were analyzed using ANOVA with Tukey's HSD *post hoc* test. Non-normally distributed data were analyzed using an independent samples Kruskal–Wallis test for multiple groups. All paired analysis was performed using Wilcoxon's signed rank test.

## Results

Microstructural analysis of the bone in the proximal tibial metaphysis comparing unoperated SHAM versus OVX animals confirmed significant bone loss in the OVX animals (Supplementary Table S1). Postoperative radiographs showed no complications in any of the animals. One animal in the CS/HA group died at the 4-week micro-CT imagining session due to anesthesia complication. One sample from the CS/HA + ZA group was excluded from the 4-week micro-CT analysis due to movement artifacts during the *in vivo* scans. During the 8-week micro-CT analysis, one sample each from the empty group and the CS/HA + ZA group were excluded due to artifacts during imaging. Initially, *n* = 9/group were used for mechanical testing, but one sample in each of the empty, CS/HA, and CS/HA + ZA + rhBMP-2 groups had to be excluded due to fractures occurring in the femoral neck canal during the adjustment of the compression device. Thus, the sample sizes for the biomechanical testing were *n* = 8 for the empty, CS/HA, and CS/HA + ZA + rhBMP-2 groups and *n* = 9 for the CS/HA + ZA group.

### *In vivo* micro-CT at 4 weeks

**FIG. 3.**
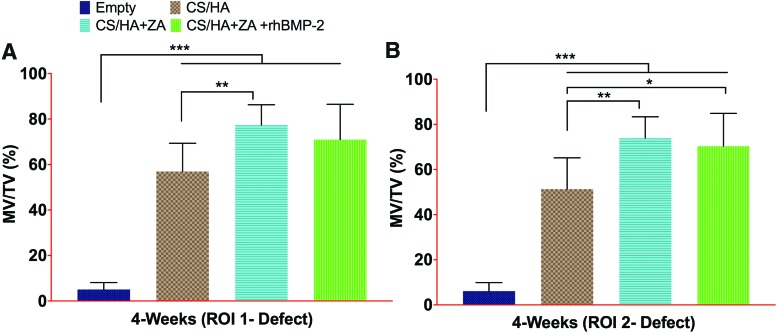
Micro-CT results from 4 weeks post surgery. **(A** and **B)** The MV/TV% in ROI 1 and ROI 2, respectively, from the *in vivo* scans at 4 weeks post surgery. *p < 0.05, **p < 0.01, and ***p < 0.001. Color images available online at www.liebertpub.com/tea

In ROI 1, that is, the entire drilled region, the MV/TV in all CS/HA-treated groups was higher than in the empty group (*p* < 0.001; Fig. 3A). The MV/TV in the CS/HA + ZA group was also higher than in the CS/HA group (*p* < 0.01). No differences were seen in the MV/TV between the CS/HA and CS/HA + ZA + rhBMP-2 groups. Furthermore, no differences were observed between the CS/HA + ZA and CS/HA + ZA + rhBMP-2 group. Similar results were seen in ROI 2 (femoral neck canal), with the only difference being that the CS/HA + ZA + rhBMP-2 group was higher than the CS/HA group (Fig. 3B; *p* < 0.05).

### *Ex vivo* micro-CT at 8 weeks

**FIG. 4.**
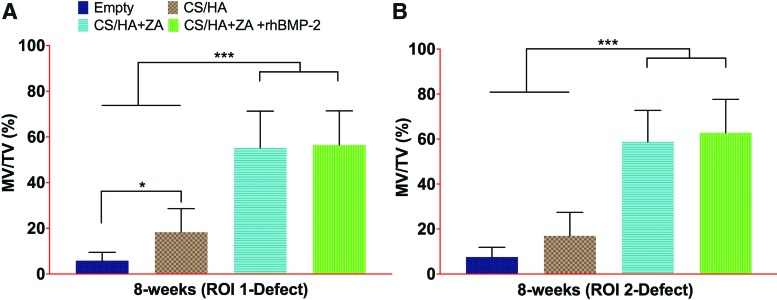
Micro-CT results from 8 weeks post surgery. **(A** and **B)** The MV/TV% of the defect leg in ROI 1 and ROI 2, respectively, 8 weeks post surgery. *p < 0.05, and ***p < 0.001. Color images available online at www.liebertpub.com/tea

For ROI 1, the MV/TV was higher in the CS/HA group (*p* < 0.05) and in the CS/HA + ZA and CS/HA + ZA + rhBMP-2 groups (*p* < 0.001) compared to the empty group (Fig. 4A). Both the CS/HA + ZA group and the CS/HA + ZA + rhBMP-2 group had significantly higher MV/TV compared to the CS/HA group (*p* < 0.001). No significant differences were seen between the CS/HA + ZA and CS/HA + ZA + rhBMP-2 groups. In ROI 2, no differences were seen in MV/TV between the empty group and the CS/HA group (Fig. 4B). The MV/TV in both the CS/HA + ZA and the CS/HA + ZA + rhBMP-2 groups was higher than both in the empty group and the CS/HA group (*p* < 0.001). Similar to ROI 1, no significant differences in the MV/TV% was seen between the CS/HA + ZA group and the CS/HA + ZA + rhBMP-2 group.

Irrespective of the treatment on the defect side, no significant differences in MV/TV was seen in the contralateral leg in ROI 1 or ROI 2 (Table 1).

**Table 1.** Micro-Computed Tomography, Biomechanical Testing Data, and Statistical Analysis

		*Treatment groups*						
*Method*	*Parameter*	*Empty (A)*	*CS/HA (B)*	*CS/HA+ZA (10 μg) (C)*	*CS/HA+ZA (10 μg)+rhBMP-2 (4 μg) (D)*	*Statistical differences (between groups)*		
Micro-CT (4 weeks)	MV/TV (%) (ROI 1) defect leg	5.1 (3.1–7.0) *n* = *12*	57 (49.1–64.8) *n* = *12*	77.4 (71.5–83.4) *n* = *11*	71 (61.2–80.8) *n* = *12*	B, C, D vs. A^***^	ANOVA	
						C vs. B^**^, D vs. B, ns	*G. Howell*	
						D vs. C, ns	*Post hoc*	
	MV/TV (%) (ROI 2) defect leg	6.1 (3.7–8.5) *n* = *12*	51.3 (42.5–60.2) *n* = *12*	74 (67.7–80.3) *n* = *11*	70.4 (61.2–79.6) *n* = *12*	B,C,D vs. A^***^	ANOVA	*Wilcoxon*
						C vs. B^**^, D vs. B^*^	*G. Howell*	*Signed Rank Test(Paired Samples)*
						D vs. C, ns	*Post hoc*	
Micro-CT (8-weeks)	MV/TV (%) (ROI 1) defect leg	5.9 (3.4–8.3) *n* = *11*	18.3 (11.4–25.2) *n* = *11*	55.2 (44.5–66) *n* = *11*	56.5 (47–66) *n* = *12*	B vs. A^*^, C,D vs. A^***^	ANOVA	A_Df_ vs. A_Cl_^*^
						C, D vs. B^***^	*G. Howell*	B_Df_ vs. B_Cl_ ns
						D vs. C, ns	*Post hoc*	C_Df_ vs. C_Cl_^**^
								D_Df_ vs. D_Cl_^**^
	MV/TV (%) (ROI 1) contralateral leg	12.4 (8.6–16.2) *n* = *11*	14.5 (7.6–21.4) *n* = *11*	11.6 (8.3–14.8) *n* = *11*	16.8 (10.8–22.9) *n* = *12*	No statistically significant differences	ANOVA	
							*Tukey*	
							*HSD*	
	MV/TV (%) (ROI 2) defect leg	7.6 (4.7–10.5) *n* = *11*	17 (10–24) *n* = *11*	58.8 (49.5–68.2) *n* = *11*	62.8 (53.4–72.2) *n* = *12*	B vs. A, ns; C, D vs. A^***^	ANOVA	A_Df_ vs. A_Cl_^*^
						C, D vs. B^***^	*G.Howell*	B_Df_ vs. B_Cl_ ns
						D vs. C, ns	*Post hoc*	C_Df_ vs. C_Cl_^**^
								D_Df_ vs. D_Cl_^**^
	MV/TV (%) (ROI 2) contralateral leg	16.5 (11.5–21.5) *n* = *11*	18 (10.7–25.4) *n* = *11*	15.5 (11.4–19.6) *n* = *11*	21.5 (14.6–28.5) *n* = *12*	No statistically significant differences	ANOVA	
							*Tukey*	
							*HSD*	
Mechanical testing (8-weeks)	Peak force (N) defect leg	131.2 (117.7–144.6) *n* = *8*	136 (127–144.9) *n* = *8*	145.4 (128.3–162.5) *n* = *9*	151.2 (137.4–164.9) *n* = *8*	No statistically significant differences	K-Wallis	A_Df_ vs. A_Cl_ ns
							Multiple sample test	B_Df_ vs. B_Cl_^*^
								C_Df_ vs. C_Cl_ ns
								D_Df_ vs. D_Cl_ ns
	Peak force (N) contralateral leg	135.7 (119.5–152) *n* = *8*	149.4 (137.1–161.6) *n* = *8*	144.5 (135.4–153.6) *n* = *9*	145.9 (133.8–158) *n* = *8*	No statistically significant differences	K-Wallis	
							Multiple sample test	
	Ratio (defect/contralateral)	0.98 (0.85–1.11) *n* = *8*	0.91 (0.86–0.96) *n* = *8*	1 (0.92–1.09) *n* = *9*	1.04 (0.92–1.17) *n* = *8*	No statistically significant differences	ANNOVA	
							*Tukey*	
							*HSD*	

The subscript Df indicates the defect leg and Cl is contralateral. Data in parentheses represent the lower and upper bounds of the 95% confidence interval. Italicized numbers show the sample size for each evaluation method and each group. Statistical significance is set at: ^*^p < 0.05, ^**^p < 0.01, and ^***^ *p* < 0.001.

A, empty; B, CS/HA; C, CS/HA + ZA; D, CS/HA + ZA + rhBMP-2; CS, calcium sulphate; HA, hydroxyapatite; ZA, zoledronic acid; rhBMP-2, recombinant human bone morphogenic protein-2; micro-CT, micro computed tomography; ROI, region of interest; ns, nonsignificant differences.

When comparing the defect leg to the contralateral leg, the MV/TV in both ROI 1 and ROI 2 was significantly lower in the defect leg of the empty group (*p* < 0.05; Table 1). No changes in the MV/TV in either ROI 1 or ROI 2 was seen between the defect and the contralateral legs when CS/HA was implanted (Table 1). The MV/TV of the CS/HA + ZA group and the CS/HA + ZA + rhBMP-2 group in the defect leg was higher than their respective contralateral legs (*p* < 0.01) for both ROI 1 and ROI 2 (Table 1).

### *Ex vivo* biomechanical testing of the femoral neck

**FIG. 5.**
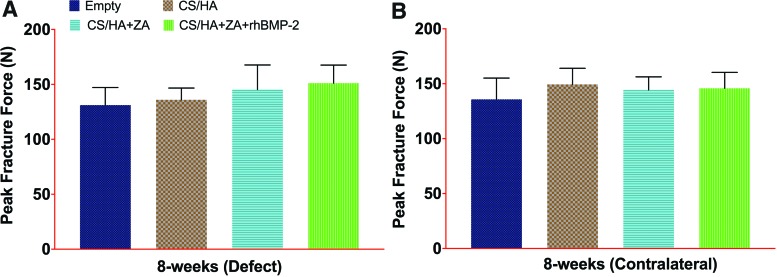
Biomechanical analysis 8 weeks post surgery. **(A** and **B)** The peak force to fracture in the defect leg and the contralateral leg, respectively, 8 weeks post surgery. No statistically significant differences between the groups were found. Color images available online at www.liebertpub.com/tea

No difference was found in the peak force to fracture in the biomechanical testing for either the defect or contralateral control legs (Fig. 5). However, when a paired analysis between the defect and contralateral controls was performed, the defect leg in the CS/HA group had significantly lower average peak force to fracture (*p* < 0.05; Table 1). In all other groups, no significant differences in the peak force to fracture between defect and contralateral legs could be observed (Table 1).

### Location of fracture

**FIG. 6.**
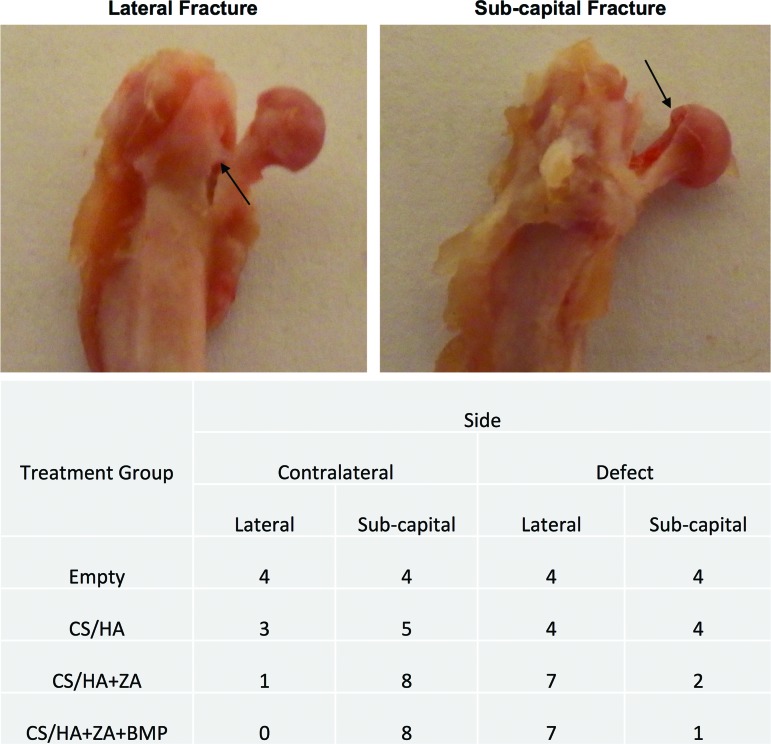
Biomechanical testing and location of fracture. *Top panel* shows fracture classification based on the location of the fracture: *Arrow* in the *left* image points to the lateral fracture and the *arrow* in the *right* image points at the sub-capital fracture. Table in the *bottom panel* shows the number of fractures of each type in different treatment groups and tested legs. Color images available online at www.liebertpub.com/tea

Fractures were classified as lateral or sub-capital, and a description of fracture location for each treatment group is provided in Figure 6.

### Qualitative histology

**FIG. 7.**
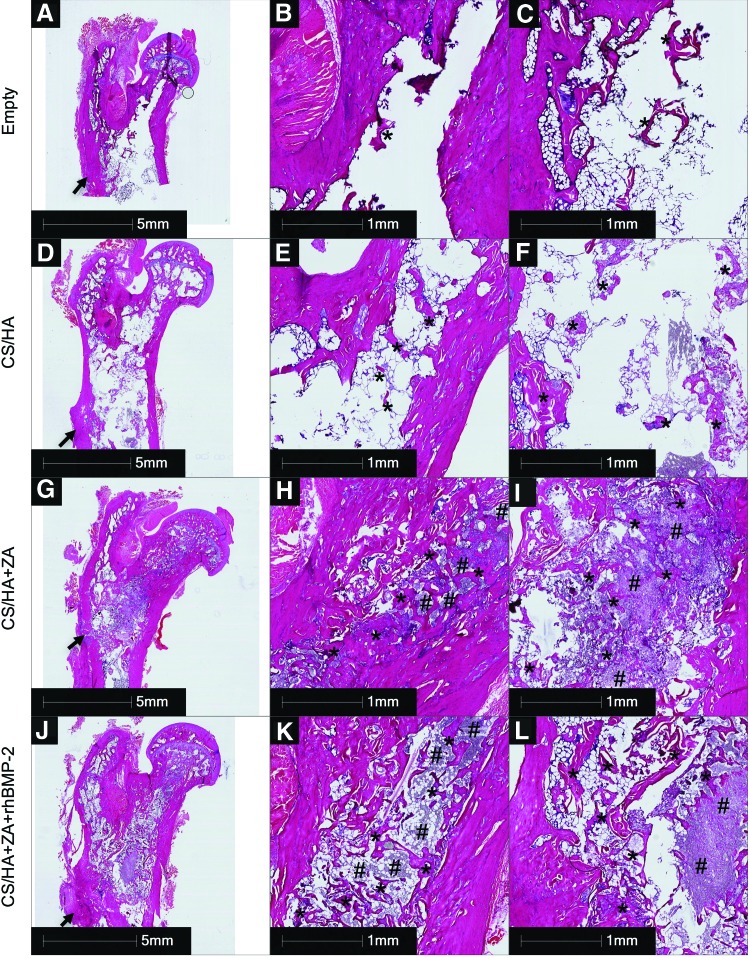
Representative histology 8 weeks post surgery. H&E-stained specimen from the empty group at low magnification **(A)**, in the femoral neck canal **(B)**, and in the femoral region **(C)**. H&E-stained specimens from the CS/HA **(D–F)**, CS/HA + ZA **(G–I)**, and CS/HA + ZA + rhBMP-2 **(J–L)** groups. **(D, G,** and **J)** A low-magnification overview of the samples. **(E, H,** and **K)** Bone formation in the femoral neck canal. **(F, I,** and **L)** Bone formation in the femoral region. The *arrows* in **(A, D, G** and **J)** point at the point of entry of the drill bit in the cortex. *Bone formation. #Remnants of the CS/HA material in the form of HA particles. ZA, zoledronic acid; rhBMP, recombinant human bone morphogenic protein-2; H&E, hematoxylin and eosin. Color images available online at www.liebertpub.com/tea

The empty group showed no signs of bone formation in the defect region, that is, neither in the femoral neck canal region nor in the femoral region (Fig. 7A–C). In the CS/HA-treated group (Fig. 7D–F), small amounts of new bone could be seen in the femoral neck canal (Fig. 7E). However, it was noted that the material had migrated toward the femoral region, as seen by HA crystals in the medullary canal (Fig. 7F). Signs of new bone formation were more pronounced in this region. In both the CS/HA + ZA (Fig. 7G–I) and CS/HA + ZA + rhBMP-2 (Fig. 7J–L) groups, more new bone could be seen both in the femoral neck canal and toward the femoral region. In many areas, bone could be seen growing around the HA particles. Furthermore, remnants of the material could also be seen in both cases. The bone formed in the CS/HA + ZA + rhBMP-2 group appeared to be more remodeled, characterized by the presence of more remodeled and less compact trabecular islands throughout the defect area. Contrary to this, the bone formed in the CS/HA + ZA groups was more densely organized.

## Discussion

This study evaluated bone formation in a novel femoral neck model of bone regeneration following implantation of an injectable bio ceramic using OVX rats to mimic postmenopausal osteoporosis. In osteoporosis, the femoral neck is of the highest clinical interest for local bone augmentation and fracture prevention. Several studies have demonstrated the effect of OVX on bone loss at various anatomical sites in rats.^23,25,26^ The Food and Drug Administration has also approved the OVX rat model as a model to study both pharmacological and material testing for osteoporosis. The strain, age at OVX and age at surgery, affecting trabecular bone loss, were chosen based on a previously published study by Li *et al.* who performed OVX on 3-month-old Sprague–Dawley rats. A significant reduction in the amount of trabecular bone was reported 90 days post OVX, reaching a plateau at approximately 9 months post OVX.^23^ The effect of OVX on the cortical bone was first noted only 1 year post OVX. The surgical protocol described in this study has not been used before in OVX rats. However, a similar model has been reported earlier in an osteoporotic model of cynomolgus monkeys.^27^ Seeherman *et al.* injected calcium phosphate biomaterial intraosseously with or without rhBMP-2 (0.75 mg/animal) into the femoral neck of OVX monkeys and demonstrated an increase in trabecular volume, cortical thickness, as well as the maximum bending force in the femoral neck 6 months post operation, the maximum duration of their study.^27^

*In vivo* micro-CT results at 4 weeks showed a significant increase in the MV/TV of all CS/HA-treated groups when compared to the empty group. However, the intergroup differences in the CS/HA treatment, irrespective of the bioactive factor added, were less than the differences at 8 weeks, which could be attributed to the high density of the biomaterial, interfering with the micro-CT quantifications at 4 weeks. An earlier study of the degradation of the CS/HA biomaterial in an ectopic muscle pouch model showed that the CS phase takes around 6 weeks to resorb, while the HA remained through the 8 weeks of the study.^20^ This slow degradation of the material during the initial 4 weeks might explain the small differences between the MV/TV in all the CS/HA-treated groups at 4 weeks. At 8 weeks, when all CS phase has been resorbed, the difference in the MV/TV in the CS/HA groups increased. The addition of CS/HA in the defect led to a significant increase in MV/TV% in ROI 1 compared to the empty group (*p* < 0.05). The choice of augmenting the CS/HA biomaterial with rhBMP-2 and ZA was obvious due to osteoinductive properties of rhBMP-2 and anti-resorptive properties of ZA. Both molecules have been extensively used in preclinical models mimicking osteoporosis.^28–30^ Mathavan *et al*. recently showed that when using BMP-7 in an osteoporotic rat femoral osteotomy model, a 100% union rate was achieved compared to 56% in untreated control rats. This study also demonstrated a slightly increased effect of BMP-7 in osteoporotic animals compared to the nonosteoporotic rats.^29^ Similarly, Gao *et al.* used a HA-coated titanium implant with a surface coating of ZA in the tibiae of OVX rats. They found that around an eight times higher pushout force was required for implants in the ZA-treated group compared to the untreated group.^30^

The results from the 8-week micro-CT experiments corroborate well with the published results. The addition of rhBMP-2 and ZA increased MV/TV in both defect ROIs significantly compared to the empty and the CS/HA group alone. Also, noteworthy is that the micro-CT results between the CS/HA + ZA and the CS/HA + ZA + rhBMP-2 groups did not show any significant differences. This suggests that controlled and local delivery of ZA alone in a bony environment may lead to significant bone formation. This is a debatable finding, and while some *in vitro* studies have suggested ZA at very low concentrations can induce osteogenic differentiation of mesenchymal stem cells,^31^ others described ZA-inducing apoptosis in human osteosarcoma cells, MG-63.^32^ Recent studies have shown a positive effect by local delivery of ZA alone in a bone defect *in vivo.*^21,22,30^ Whether these effects are only anti-catabolic, meaning that ZA prevents resorption of bone that would form irrespective of treatment with a bioactive molecule or if endogenous growth factors including BMPs act synergistically with ZA, is unknown. Further mechanistic studies are required to establish the ability of ZA to cause increased net bone formation.

An important aspect of this study was to ensure the local delivery of both ZA and rhBMP-2. The aim was to circumvent the side effects of systemic administration of ZA and targeted spatiotemporal delivery of rhBMP-2, respectively. Different polymeric systems have been used for this task.^11,33^ However, the noninjectability of these materials requires invasive surgical methods. The CS/HA biomaterial provides an injectable platform for delivery of both bioactive molecules and with a minimally invasive approach. The comparisons from the micro-CT and biomechanical testing of the contralateral sides clearly indicated that none of the treatments has a systemic effect on the contralateral femur, which is a major goal of local delivery. Furthermore, paired analysis of MV/TV of the CS/HA + ZA and CS/HA + ZA + rhBMP-2 groups showed a significant increase in the defect leg compared to the contralateral leg, which emphasizes the efficacy of local delivery in this model. One might argue that the MV/TV is not an accurate measure of bone formation when the bone has been treated with a radiopaque biomaterial such as CS/HA. However, representative histology from the CS/HA + ZA and CS/HA + ZA + rhBMP-2 groups confirmed abundant amounts of regenerated viable trabecular bone in the femoral neck canal region as well as in the trochanteric region (Fig. 7), thereby strengthening the micro-CT quantifications.

As an indirect translation of significant bone regeneration in the femoral canal of CS/HA + ZA- and CS/HA + ZA + rhBMP-2-treated animals, one would also expect an increased mechanical strength. However, the biomechanical testing did not show significant differences between any of the treatment groups on either of the legs. A mechanical testing protocol described earlier by Hessle *et al.* was used.^24^ In this study, a critical size trabecular bone defect was created in the femoral neck.^34^ It is speculated that most of the compressive mechanical strength (>90%) of the femoral neck in humans is provided by the cortical bone, whereas the trabecular bone contributes <10%.^34^ The trabecular bone contributes more to the shear strength, which is not tested in the current compression testing setup. The femoral neck and the intertrochanteric region have a larger proportion of trabecular bone than the subtrochanteric region, and the trabecular bone is generally weaker than the cortical bone. Another important aspect to emphasize is the age of the animals used in this study. Li *et al*. followed the development of osteopenia-like features in the proximal femur of OVX rats over 1 year and only noticed a significant reduction in the cortical width after the 1-year time point.^23^ In this study, only 16 weeks passed post OVX, and one can assume that the progression of cortical thinning at this time point is negligible compared to the non-OVX rats. Potentially, the biological need to guide cortical regeneration in an already healthy cortex at this time point is absent from a bone homeostasis perspective. Whether a longer follow-up post surgery, older age at OVX, or a different mechanical testing protocol would change the biomechanical results are only speculative at the moment, and further experiments will be required to verify these.

The absence of a systemic ZA group is a limitation of the study, since such a group could potentially have illuminated the role of local delivery of ZA in this animal model. Furthermore, CS/HA + rhBMP-2 was also not used without ZA. However, based on previous experience, the authors were confident about the efficacy of local ZA delivery using the CS/HA biomaterial.^16,22^ These studies also indicated that the CS/HA biomaterial produces significantly higher volume of bone when combined with both rhBMP-2 and ZA compared to only rhBMP-2, and thus the number of experimental animals was reduced based on the guidelines of the 3Rs Principle.^35^ The age of the animals at OVX, as well as the time to wait post OVX, might be considered to be rather premature, and other studies have suggested different ages.^23,25,36^ The age most likely to induce osteoporosis-like characteristics in the trabecular bone of the proximal femur of rats reported earlier was chosen.^23^ Also, the interest in this study was in trabecular bone regeneration, and slightly younger rats would have possessed more responsive stem/progenitor cells compared to older rats.^37^ The quantitative results in this study are based on micro-CT imaging, but it is important to keep in mind that separating new bone formation and ceramic biomaterial remnants is very challenging. For this reason, the results present quantifications of bone/material composite with a high density. Despite this, the representative histology findings support the micro-CT findings well. Further, this study failed to show whether the bioavailability of local ZA and rhBMP-2 is only early on or prolonged in this model. However, according to an ongoing study, a strong interaction of ZA with the CS/HA biomaterial and a sustained release of rhBMP-2 over a period of 4 weeks *in-vivo* has been confirmed. Based on these results, it is speculated that ZA is locally available for a long period of time, while rhBMP-2 could possibly have been released completely after 8 weeks.^38^ It was also not possible to elucidate the mechanism of small amounts of released ZA reuptake by surrounding bone or other skeletal sites in this study, though no significant effects were seen on the contralateral side. ^14^C Radiolabeled ZA might be able to elucidate a complete pharmacodynamics of ZA from the CS/HA biomaterial when injected in the femoral neck canal. Finally, this study did not elucidate if the addition of ZA has any impact on CS/HA resorption for which long-term studies would be required.

## Conclusions

In conclusion, the model developed to study the process of bone regeneration in the femoral neck canal of OVX rats in this study is novel and promising for future research. The CS/HA biomaterial combined with ZA or both rhBMP-2 and ZA led to significantly enhanced bone regeneration in the femoral neck canal of osteoporotic rats in this model, as seen from micro-CT and histology. CS/HA biomaterial alone was superior to the empty group in the entire defect region. Moreover, these results did not translate into an increased mechanical strength in the treated legs compared to the untreated legs. Long-term studies with increased time post OVX and possibly a new biomechanical testing protocol are required to establish whether this combination can be used as a preventive treatment for osteoporotic fractures.

## Supplementary Material

Supplemental data
