# Supplementary material for: Calcium Sulphate/Hydroxyapatite Carrier for Bone Formation in the Femoral Neck of Osteoporotic Rats
Source: Tissue Eng Part A. 2018 Dec 3;24(23-24):1753–64. doi: 10.1089/ten.tea.2018.0075 (PMC6302674; doi:10.1089/ten.tea.2018.0075)
Supplement: Supplemental data [file Supp_Data.pdf]

Supplementary Data

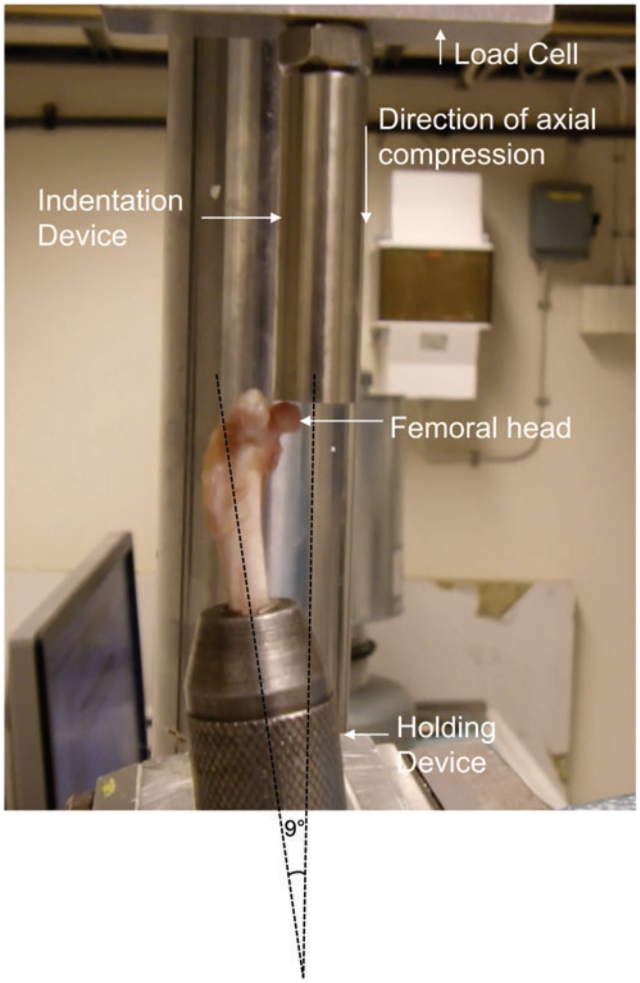

SUPPLEMENTARY FIG S1. Experimental setup for biomechanical testing.

SUPPLEMENTARY TABLE S1. COMPARISON OF BONE MICROSTRUCTURAL PARAMETERS IN SHAM VERSUS OVX ANIMALS

	Parameter			
Group	*BV/TV, %*	*Trabecular number (Tb.N), 1/mm*	*Trabecular separation (Tb.Sp), mm*	*Trabecular thickness (Tb.Th), mm*
SHAM (*n*=3)	17.2±8.3	0.039±0.015	16.5±4.7	4.2±0.6
OVX (*n*=4)	4.0±2.8	0.007±0.005	67.0±27.3	5.3±0.3
